# Clinical outcomes after revision knee arthroplasty due to periprosthetic joint infection: A single‐centre study of 359 knees at a high‐volume centre with a minimum of one year follow‐up

**DOI:** 10.1002/ksa.12762

**Published:** 2025-07-07

**Authors:** Rasmus Liukkonen, Meeri Honkanen, Eerik Skyttä, Antti Eskelinen, Matti Karppelin, Aleksi Reito

**Affiliations:** ^1^ Coxa Hospital for Joint Replacement and Faculty of Medicine and Health Technology Tampere University Tampere Finland; ^2^ Department of Internal Medicine Tampere University Hospital Tampere Finland

**Keywords:** DAIR, one‐stage revision, periprosthetic joint infection, surgical outcomes, two‐stage revision

## Abstract

**Purpose:**

Decisions on the treatment of periprosthetic joint infection (PJI) are typically guided by established algorithms. However, as these algorithms often lack substantial supporting evidence, this study aimed to evaluate 1‐year survival rates and compare different surgical approaches.

**Methods:**

In this single‐centre retrospective cohort study, all revisions of the knee due to PJI with at least 1 year of follow‐up performed between January 2008 and September 2021 were identified. In total, 141 debridement, antibiotics, and implant retentions (DAIRs), 98 one‐stage, and 120 two‐stage revisions were performed. Infections were classified as early, acute hematogenous, or chronic infections. Survival was calculated using the Kaplan–Meier method and the cumulative incidence function. Predictors of outcomes were examined with Fine‐Gray regression and Cox proportional hazards regression, and subdistribution hazard ratios (sdHR) and adjusted hazard ratios (aHR) with 95% confidence intervals (CIs) were calculated.

**Results:**

At 1‐year follow‐up, 23% (CI 19%–27%) of patients had undergone a reoperation, and 4% (CI 2%–6%) had died. The risk of reoperation was largest after two‐stage revision (28%, CI 20%–36%) and smallest after one‐stage revision (15%, CI 9%–23%). For every infection type, the failure rates at one‐year follow‐up favoured one‐stage revision over two‐stage revision. Higher ASA‐scores increased the risk of death (aHR 1.7, CI 1.1–2.5 per one‐unit increase).

**Conclusion:**

The risk of failure after one‐year follow‐up is high after revision for periprosthetic joint infection. The lowest risk was observed after one‐stage revision; however, this may partly reflect patient selection, as one‐stage revision may not be suitable for all patients.

**Level of Evidence:**

Level III, retrospective comparative study.

**Abbreviations**
aHR: adjusted hazard ratio
ASA: American Society of Anesthesiology
BMI: body mass index
CCI: Charlson's Comorbidity Index
CI: confidence interval
DAIR: debridement, antibiotics, and implant retention
EHR: Electronic Health Records
ICD‐10: International Classification of Diseases 10th revision
IQR: interquartile range
MSIS: Musculoskeletal Infection Society
PJI: periprosthetic joint infection
RCT: randomised controlled trial
SD: standard deviation
sdHR: sub‐distribution hazard ratios

## INTRODUCTION

Treatment decisions of periprosthetic joint infections (PJIs) after total knee arthroplasty have traditionally been based on treatment algorithms [8]. However, the evidence behind these algorithms is vague, and hence no universal consensus on the optimal treatment for PJI exists [2, 11].

In cases where implant retention is not possible, components are traditionally removed and replaced in either a one‐stage operation or in two separate operations with a period of spacer prosthesis in between (two‐stage revision) [8]. In recent years, however, a so‐called ‘1.5‐stage exchange arthroplasty’, where the second stage of the originally intended two‐stage operation is cancelled due to satisfactory outcome from the first stage, and the articulating spacer from the first stage is retained in the joint, has become a viable treatment option [7, 16, 23]. Moreover, when compared to two‐stage revision, reinfection rates from the one‐stage revisions have been reported to be acceptable [7, 16, 23]. As two‐stage revision results in increased morbidity and economic burden on the health care system when compared to one‐stage revision, more research is needed to identify those patients who would benefit the most from two‐stage revision [17].

To our best knowledge, no prior clinical study has examined the outcomes between different surgical strategies in a single‐centre setting. In the present study, the aim was to assess: (1) What is the short‐term survival rate after PJI revision? and (2) How do the outcomes of the surgical strategies differ? The hypothesis was that the results from one‐stage revisions would be superior to those from two‐stage revisions.

## METHODS

### Study design

**Figure 1:**
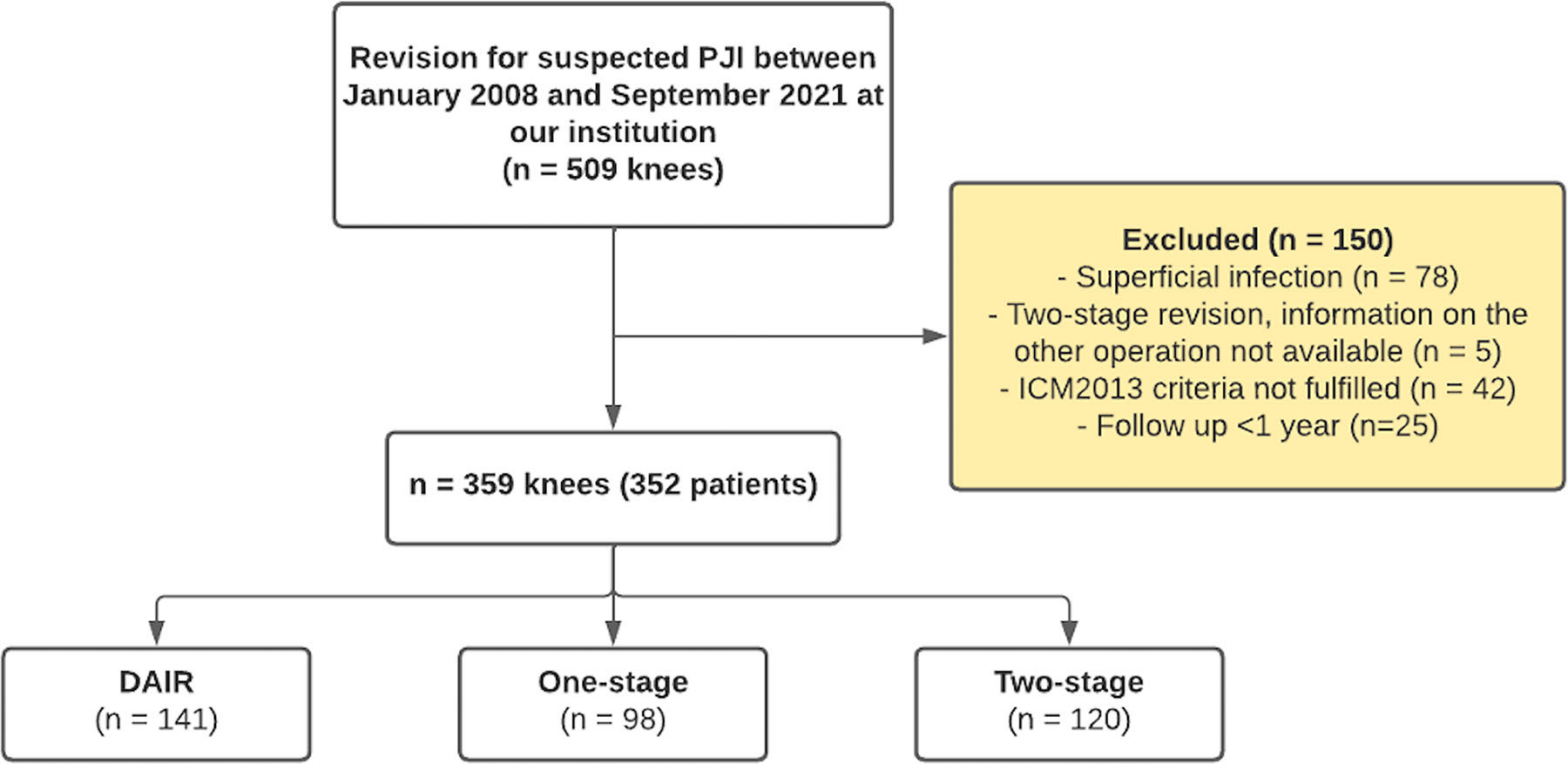
Flowchart of the patients at our institution between January 2008 and September 2021. DAIR, debridement, antibiotics, and implant retention; PJI, prosthetic joint infection.

This retrospective cohort study analysed knee revision surgeries for periprosthetic joint infection performed at a single institution. The study period began on 1 January 2008. Patient data was accessed for research purposes on 12 September 2021, which also marked the end of the study period. To identify the surgeries, the ICD‐10 (International Classification of Diseases 10th revision) code T84.5 was searched (infection and inflammatory reaction due to an internal joint prosthesis). In addition, the 2013 International Consensus Diagnostic Criteria were applied to confirm the diagnosis of PJI [26]. If the criteria were not met, the patient was excluded from the study. Superficial wound infections and two‐stage operations where information about the initial surgery was unavailable were also excluded. Only the first revisions performed specifically for PJI were included. If a patient had PJI in both knees, each knee was analysed as a separate operation. In addition, patients with less than one year of follow‐up were excluded (Figure 1).

### Data sources

Patient data were obtained from the institution's electronic data lake and electronic health records (EHR). The following patient demographics were collected: age, sex, body mass index (BMI), American Society of Anesthesiology (ASA) classification, and comorbidities. Charlson's comorbidity indexes (CCI) were retrospectively calculated for each patient. In addition, the date of the primary surgery, the date of the most recent non‐infectious operation on the same joint, and the date the symptoms began before revision surgery were collected. Information on the presence of a fistula and microbiological findings obtained from tissue specimens during the surgery was also collected from the EHRs. All microbiology analyses were performed in the accredited microbiology laboratory of the local university hospital. In accordance with Finnish legislation, no ethics committee is required for the research of the registry data. Approval to access the hospital's database was granted by the Institutional Review Board of the Pirkanmaa Hospital District (R22674). Since the study utilised retrospective registry data, informed consent was not required as the patients were not contacted. As we included all the surgeries that met the inclusion criteria, no sample size calculation was performed.

The surgeries were categorised into one of the following three categories: DAIR, one‐stage revision, or two‐stage revision. If the planned second stage was not performed due to a satisfactory outcome from the first‐stage operation with an articulating spacer (1.5‐stage exchange arthroplasty [7]), the surgery was categorised as one‐stage revision (*n* = 20), as suggested by the Musculoskeletal Infection Society (MSIS) [6]. To reflect the pathogenesis of PJI and to produce results that are applicable in a clinical setting, the infections were classified as early (≤ 90 days from the previous surgery), acute hematogenous (> 90 days from the previous surgery AND < 28 days of symptoms) and chronic infections (> 90 days from the previous surgery AND ≥ 28 days of symptoms). Chronic infections, commonly known as low‐grade infections, were categorised so, because they are usually caused by less virulent pathogens, and hence are less aggressive than acute infections, which are commonly caused by more virulent and more aggressive pathogens [10, 24].

The treatment decisions were based on international consensus recommendations, where early and hematogenous infections are preferably treated with either DAIR or one‐stage revision [8, 18]. For chronic infections, two‐stage or one‐stage revision was the preferred treatment method. In addition, each of the knees was treated according to the up‐to‐date consensus recommendations. At our institution, the proportion of one‐stage revisions has increased rapidly during the last decade, with almost all PJIs now being managed with either DAIR or one‐stage revision [14].

Postoperative antimicrobial treatments were designed by infectious‐disease specialists. Since 2014, the usual practice has been to administer postoperative antibiotic therapy intravenously for 2 weeks followed by four weeks of oral therapy, regardless of the surgical modality. Between 2008 and 2014, the total duration of treatment may have been up to 3 months. However, parenteral treatment very rarely exceeded four weeks when highly bioavailable oral treatment was used. Antibiotics are discontinued after the second stage operation when intraoperative cultures are negative and there is no patient‐specific indication for prolonged suppressive antibiotic treatment. In staphylococcal infections, a rifampin‐based combination was used when not contraindicated (drug interactions or high risk for adverse reactions) except in two‐stage revisions with no foreign material left in situ.

### Primary and secondary outcomes

Follow‐up started from the day of the revision surgery due to PJI and ended when the patient was lost to our institution's regular follow‐up programme (e.g., death or patient moved to another area) or on the date of data collection, whichever came first. Reoperation was defined as a new surgical procedure on the previously operated joint. In addition, the outcomes of the revision surgeries were categorised according to the MSIS categorisation scheme [6]. In cases of two‐stage revision, the first operation was the starting point for the follow‐up period, as recommended by the MSIS [6]. In survival analyses, our primary outcome was reoperation due to any reason (MSIS tiers 3A–3E), as it has been suggested that aseptic revision performed within 1 year from the initial surgery for the treatment of PJI represents a secondary failure due to PJI [6]. Death from any cause (MSIS tiers 4A&4B) was considered to be a competing risk because we did not have access to the cause of death, and it was not possible, therefore, to clarify whether the death was PJI‐related [4].

### Statistical analysis

Means with standard deviations (SD) are presented for normally distributed variables, and medians with ranges or interquartile ranges (IQR) for variables with non‐Gaussian populations. Cumulative incidences of reoperations and deaths were calculated as described by Scrucca et al. [19]. The risk of any‐cause failure was calculated using the Kaplan–Meier estimator. Results are presented with 95% confidence intervals (CIs).

A Fine‐Gray regression model was used to identify potential predictors for reoperation or death, as the model has been reported to be more accurate than cause‐specific Cox regression when estimating a single patient's clinical prognosis [1]. However, the cause‐specific Cox models for both reoperation and death were calculated, and the results from those analyses are also presented [12]. In the Cox models, the proportional hazards assumptions were tested using Schoenfeld's residuals, and the assumptions were not violated in any tested model.

To assess the effect of confounding factors and to predict the outcomes more accurately, multivariable analyses were performed. Due to the many possible predictors of outcome, variable selection processes were performed (Supporting Information: Figure 1). First, global models were formed based on known risk factors and clinically relevant factors (Supporting Information: File 1). The variables included in these global models were selected for the final Fine‐Gray regression models using backward elimination with a significance level of 0.157 (AIC selection). For the cause‐specific Cox regression models, the variables were selected based on the combination of backward elimination with a significance level of *p* < 0.10. Thereafter, model stabilities were assessed by bootstrap stability investigation with 200 repetitions. Based on these two investigations, the final variables for the regression analyses were selected. Results from multivariable analyses are presented with either adjusted sub‐distribution hazard ratios (sdHR) or adjusted hazard ratios (aHR). All analyses were performed using R (version 4.1.2; R Foundation for Statistical Computing, Vienna, Austria).

## RESULTS

### Patient demographics

A total of 359 revisions (352 patients) with at least one‐year of follow‐up were identified. Of these, 141 (39%) were DAIRs, 98 (27%) were one‐stage revisions, and 120 (33%) were two‐stage revisions (Figure 1). Most of the PJIs were early (39%, *n* = 139) or acute hematogenous (38%, *n* = 136) infections. Further details on patient demographics are presented in Table 1.

**Table 1:** Characteristics of patients with PJI and preoperative risk factors stratified by the surgical technique.

	DAIR (*n* = 141)	One‐stage (*n* = 98)	Two‐stage (*n* = 120)
Patient characteristics			
Women, *n* (%)	66 (46.8)	49 (50)	67 (55.8)
Age, median (IQR), y	70 (63–77)	74 (66–82)	71 (62–79)
BMI, mean (SD)	30.7 (5.8)	29.1 (5.5)	31.2 (6.2)
CCI, median (range)	3 (0–7)	3 (0–8)	3 (0–6)
ASA‐class, *n* (%)			
1	3 (2.1)	3 (3.1)	2 (1.7)
2	31 (22)	12 (12.2)	25 (20.8)
3	93 (66)	64 (65.3)	77 (64.2)
4	10 (7.1)	16 (16.3)	12 (10)
5	1 (0.7)	1 (1)	0
NA	3 (2.1)	2 (2)	4 (3.3)
Co‐morbidities, *n* (%)			
Diabetes mellitus	31/123 (25.2)	15/90 (16.7)	28/109 (25.7)
Rheumatoid arthritis	12/123 (9.8)	10/89 (11.2)	17/104 (16.3)
Chronic kidney disease	4/123 (3.3)	3/89 (3.4)	2/106 (1.9)
Infection type, *n* (%)			
Early	69 (48.9)	35 (35.7)	35 (29.2)
Acute hematogenous	71 (50.4)	35 (35.7)	30 (25)
Chronic	1 (0.7)	28 (28.6)	55 (45.8)
Surgical characteristic			
Time since previous operation, median (IQR), *d*	112 (19–1336)	332 (30–1387)	332 (63–1498)
Symptom duration, median (IQR), *d*	5 (3–13)	13 (5–29)	21 (5–81)
Sinus tract, *n* (%)	49 (34.8)	28 (28.6)	38 (31.7)
Duration of the antibiotic treatment, mean (SD), wk	9.8 (4.2)	7.6 (2.4)	8.8 (3.2)
Rifampin usage, *n* (%)	63/141 (44.7)	40/93 (43)	36/106 (33.9)
Previous indication, *n* (%)			
Osteoarthritis	109 (77.3)	84 (85.7)	99 (82.5)
Aseptic revision	24 (17)	10 (10.2)	18 (15)
Other	8 (5.7)	4 (4.1)	3 (2.5)
Microbial findings, *n* (%)^a^			
*Staphylococcus aureus*	51 (36.2)	29 (29.6)	29 (24.2)
CNS	36 (25.5)	14 (14.3)	26 (21.7)
*Streptococcus* beta‐hemolyticus	22 (15.6)	11 (11.2)	9 (7.5)
Other *Streptococcus* species	5 (3.5)	1 (1)	4 (3.3)
Gram‐negative aerobic	4 (2.8)	10 (10.2)	5 (4.2)
Enterococcus species	4 (2.8)	5 (5.1)	3 (2.5)
Anaerobic	1 (0.7)	1 (1)	6 (5)
Other	3 (2.1)	2 (2)	1 (0.8)
Negative culture	30 (21.3)	36 (36.7)	42 (35)
Polymicrobial	14 (9.9)	9 (9.2)	5 (4.2)

*Note*: Infections were classified as early (≤ 90 days from the previous surgery), acute hematogenous (> 90 days from the previous surgery AND < 28 days of symptoms), and chronic infections (> 90 days from the previous surgery AND ≥ 28 days of symptoms).

Abbreviations: ASA, American Society of Anesthesiology; BMI, body mass index; CCI, Charlson comorbidity index; d, days; DAIR, debridement, antibiotics, and implant retention; IQR, interquartile range; PJI, periprosthetic joint infection; SD, standard deviation; wk, weeks; y, years.

^a^ Microbiological findings from the polymicrobial infections (*n* = 28, 31 additional pathogens) are included, and therefore the total *N* is greater than the total *N* of surgeries performed (*n* = 359).

### Outcomes after PJI revision

**Figure 2:**
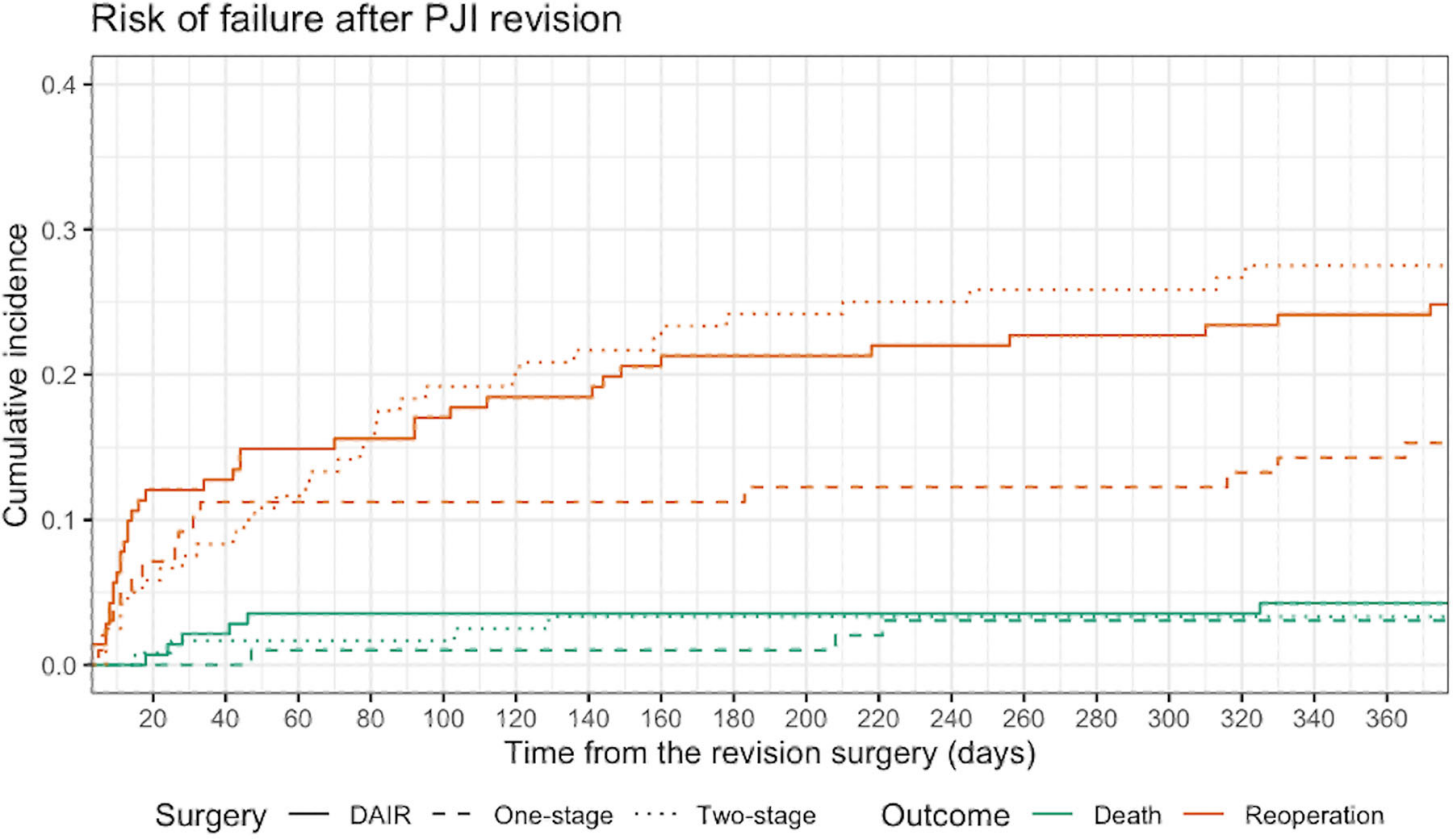
The surgical technique stratified cumulative incidences of different failure types after revision surgery for prosthetic joint infection. DAIR, debridement, antibiotics and implant retention.

The risk for reoperation was largest after two‐stage revision (28%, CI 20%–36%) and smallest after one‐stage revision (15%, CI 9%–23%). The risk for any‐cause failure was smallest after one‐stage revision (18%, CI 10%–26%) and largest after two‐stage revision (31%, CI 22%–39%). (Table 2, Supporting Information: Table 1 and Figure 2).

**Table 2:** Risk for failure after PJI revision surgery stratified by the surgical technique.

	30 Days	1 Year
Any‐cause failure		
All revisions	10.1% (7.8%–14.3%)	26.5% (21.8%–30.9%)
DAIR	14.2% (8.2%–19.8%)	28.4% (20.5%–35.4%)
One‐stage	9.2% (3.3%–14.7%)	18.4% (10.3%–25.7%)
Two‐stage	9.2% (3.9%–14.2%)	30.8% (22.1%–38.6%)
Reoperation		
All revisions	9.7% (7.0%–13.1%)	22.8% (18.6%–27.3%)
DAIR	12.1% (7.3%–18%)	24.1% (17.4%–31.5%)
One‐stage	9.2% (4.5%–15.9%)	15.3% (9%–23.2%)
Two‐stage	7.5% (3.7%–13.1%)	27.5% (19.8%–35.7%)
Death		
All revisions	1.4% (0.5%–3.1%)	3.6% (2.0%–5.9%)
DAIR	2.1% (0.6%–5.6%)	4.3% (1.7%–8.5%)
One‐stage	0%	3.1% (0.8%–8%)
Two‐stage	1.7% (0.3%–5.3%)	3.3% (1.1%–7.7%)

*Note*: Any‐cause failure rates were calculated with Kaplan–Meier estimator, and cause‐specific failure rates using cumulative incidences. Results are presented with 95% confidence intervals.

Abbreviations: DAIR, debridement, antibiotics and implant retention; PJI, periprosthetic joint infection.

For every infection type, the failure rates at 1‐year follow‐up favoured one‐stage revision over two‐stage revision. However, for early infection, the results of DAIR (26%, CI 15%–36%) and one‐stage revision (31%, CI 14%–45%) were comparable (Table 3). When compared to two‐stage revision, the use of one‐stage revision was slightly associated with a decreased risk for reoperation (HR 0.5, CI 0.2–1.3) with no added mortality risk (HR 0.6, CI 0.2–2.2) after chronic infection. Furthermore, the risk for any‐cause failure was also lower (HR 0.5, CI 0.3–1.1) when one‐stage revision was performed. The results from these analyses were, however, imprecise, and confidence intervals included the zero change.

**Table 3:** Risk for any‐cause failure after PJI revision surgery stratified by the surgical technique and infection type.

	30 Days	1 Year
Early infection		
All revisions (*n* = 139)	17.3% (10.7%–23.3%)	30.9% (22.8%–38.2%)
DAIR (*n* = 69)	15.9% (6.8%–24.2%)	26.1% (15%–35.8%)
One‐stage (*n* = 35)	20% (5.6%–32.2%)	31.4% (14.2%–45.2%)
Two‐stage (*n* = 35)	17.1% (3.7%–28.7%)	40% (21.4%–54.2%)
Acute hematogenous infection		
All revisions (*n* = 136)	8.8% (3.9%–13.5%)	25% (17.4%–31.9%)
DAIR (*n* = 71)	12.7% (4.6%–20.1%)	31% (19.3%–40.9%)
One‐stage (*n* = 35)	2.9% (0%–9.2%)	8.6% (0%–17.4%)
Two‐stage (*n* = 30)	6.7% (0%–15.2%)	30% (11.5%–44.6%)
Chronic infection		
All revisions (*n* = 84)	4.8% (0.1%–9.2%)	21.4% (12.1%–29.7%)
DAIR (*n* = 1)	–	–
One‐stage (*n* = 28)	3.6% (0%–10.2%)	14.3% (0.3%–26.3%)
Two‐stage (*n* = 55)	5.5% (0%–11.3%)	25.5% (13%–36.1%)

*Note*: Failure is determined as re‐operation or death. Failure rates were calculated with the Kaplan–Meier estimator. Results are presented with 95% confidence intervals.

Abbreviations: DAIR, debridement, antibiotics and implant retention; PJI, periprosthetic joint infection.

### Risk factors for failure and predictive ability

In the selected Cox regression models, the C‐indexes were 0.64 with reoperation as the endpoint and 0.63 with death as the endpoint. The corresponding R^2^ values were 0.23 for reoperation and 0.22 for death, indicating modest predictive capability. The most important predictors of reoperation were ASA class and CCI, whereas CCI and the presence of diabetes mellitus or liver cirrhosis were the most important predictors of death (Table 4, Supporting Information: Figure 2 and Table 2).

**Table 4:** Cox proportional hazard regression hazard ratios for failure with 95% confidence intervals.

Cox cause‐specific regression (*n* = 274)	Adjusted HR (95% CI)
Hazard ratios for reoperation^a^	
Age	0.97 (0.95–0.99)
ASA‐score	0.77 (0.64–0.94)
Previous indication	1.21 (0.70–2.07)
One‐stage revision^b^	1.03 (0.54–1.95)
Two‐stage revision^b^	1.75 (1.03–2.97)
Hazard ratios for death^a^	
Age	0.97 (0.95–0.99)
ASA‐score	1.66 (1.09–2.53)
One‐stage revision^b^	1.0 (0.53–1.9)
Two‐stage revision^b^	1.75 (1.03–2.98)

Abbreviations: ASA, American Society of Anesthesiologists; CI, confidence interval; DAIR, debridement, antibiotics, and implant retention; HR, hazard ratio.

^a^ Type of the infection is adjusted for this model.

^b^ DAIR was used as the reference,

## DISCUSSION

The results of the present study reveal that one‐stage revision might be as capable as two‐stage revision for the eradication of infection with no added risk for mortality. Surprisingly, the results from two‐stage revisions were inferior for every infection type when compared with the other revision strategies.

Previous studies have demonstrated 3.7%–4.3% mortality in one‐year follow‐up after two‐stage revision for PJI of the knee [5, 15]. Our results are comparable, as 1‐year mortality in our study was 3.3% after two‐stage revision. Interestingly, mortality rates between the different revision strategies were almost similar. Mortality has previously been reported to be highest after DAIR and lowest after one‐stage revision [13, 25]. However, as most of the studies have included different types of PJIs, a direct comparison between individual studies is not possible. In the present study, we managed to compare one‐stage and two‐stage revisions within the same infection types.

For acute hematogenous infections, we observed a rather high risk for failure after DAIR. Indeed, the failure rates after one‐year follow‐up were inferior, especially when compared to the failure rates of one‐stage revision. To our best knowledge, no prior study has compared outcomes between DAIR and one‐stage revision with these types of infections [11]. Interestingly, the failure rates after two‐stage revision for acute infections were also very high. This finding might, however, have been due to the selection bias caused by the initial patient selection because most patients were managed with either DAIR or the one‐stage revision, as suggested by the international guidelines [8]. However, further research on the selection of DAIR or one‐stage revision for patients with acute infections is warranted, preferably in a randomised controlled trial setting. In addition, it is noteworthy that as the categorisation of infection types was performed retrospectively, rather than prospectively, categorisation bias might be present, further reflecting the need for future prospective trials.

For chronic infections, the results from one‐stage revisions were at least as good or even superior to those observed from two‐stage revisions, as the failure rates at one‐year follow‐up were comparable. Previously, one‐stage revision has been associated with a similar risk for re‐revision when compared to two‐stage revision [13], an association also seen in our results. However, a previous randomised controlled trial from the UK demonstrated no superiority for one‐stage revision over two‐stage revision [3]. As the patient cohort in that RCT included patients with multiple revisions, our results are not comparable. When the added costs and the stress for the patient associated with the second operation of two‐stage revision are taken into account, one‐stage revision seems to be a viable option for the treatment of PJI of the knee [3, 17]. However, as one‐stage revision might not be suitable for everyone, more research on patient selection is warranted.

The 1.5‐stage revision has previously shown very promising results, as it has been proven to be noninferior to two‐stage revision [7, 16, 20, 21, 22]. Furthermore, the 1.5‐stage revision does not exclude the possibility of explanting the spacer and implanting the definitive prosthesis in the second stage and offers a more functional outcome when compared to the traditional molded spacer prostheses because articulating spacers are used rather than the molded prostheses in the 1.5‐stage revision. In addition, the surgical technique used in 1.5‐stage revision at our institution does not differ significantly from that used in 'traditional one‐stage revision', as we use primary components for these revisions. During the 1‐year follow‐up after 1.5‐stage revision, we observed no failures, which is in line with previous results. Hence, the 1.5‐stage revision seems to be a promising alternative for the management of PJIs of the knee. However, because this concept is rather new, more research is needed to identify those patients who would benefit most from 1.5‐stage revision.

Previously, it has been reported that predicting failure after PJI revision is difficult [9]. Furthermore, the identification of modifiable predictors has been especially challenging [9]. In our analyses, a higher ASA class increased the risk for death. Moreover, this association remained after adjusting for the type of infection and the type of operation. However, the predictive ability of the selected prediction models was poor. Our results confirm that predicting failure is challenging and indicate that further research to identify modifiable preoperative factors is required.

Our study has several limitations, which are primarily due to its retrospective nature. The patient selection process for treatment strategies was not entirely conclusive, which could have introduced selection bias given the rare and diverse nature of PJI. This is a common issue in PJI research and can only be fully addressed in a prospective study. In addition, it is evident that the patients who are selected for two‐stage revision are not entirely comparable to the patients with one‐stage revision, and hence the results from the direct comparison of techniques should be interpreted with caution. However, as all patients were treated by the same surgeons at a single centre, any potential selection bias would have been minimised. Further, we did not analyse the impact of antimicrobial treatment on outcomes because some of the PJI cases were referrals, and the accuracy of the information on antimicrobial treatment was not consistent. The large sample size in our study was a significant advantage over previous PJI research, which has been mainly based on small case series or heterogeneous multicenter cohorts. However, as no sample size calculation was performed, our analyses might have been underpowered. Hence, no direct comparison between surgical strategies was performed. While a multicenter setting could result in a larger sample size, the risk for selection bias is increased when treatment decisions are not made by the same surgeons. Furthermore, due to our large sample size, we were also able to perform diverse methodological analyses to compare various treatment strategies and investigate patient‐specific factors that can serve to enhance decision‐making processes in the future.

## CONCLUSION

The risk of failure at 1‐year follow‐up remains high after revision surgery due to periprosthetic joint infection. In our cohort, the lowest risk was observed after one‐stage revision. However, this finding may reflect patient selection, as the surgical approach was not randomised and was likely influenced by clinical factors. While results after one‐stage revision appear promising, it should only be considered for carefully selected patients.

## AUTHOR CONTRIBUTIONS

**Rasmus Liukkonen**: Design of the work; data acquisition; statistical analysis; interpretation of the results; first draft of the manuscript. **Meeri Honkanen**: Design of the work; interpretation of the results; revising the manuscript; approving the final draft. **Antti Eskelinen**: Design of the work; interpretation of the results; approving the final draft; supervision. **Matti Karppelin**: Interpretation of the results; revising the manuscript; approving the final draft. **Eerik Skyttä**: Interpretation of the results; revising the manuscript; approving the final draft. **Aleksi Reito**: Design of the work; statistical analysis; interpretation of the results; revising the manuscript; approving the final draft; supervision.

## CONFLICT OF INTEREST STATEMENT

The authors declare conflicts of interest.

## ETHICS STATEMENT

In accordance with Finnish legislation, no ethics committee is required for the research of the registry data. Approval to access the hospital's database was granted by the Institutional Review Board (R22674X). Since the study utilised retrospective registry data, informed consent was not required as the patients were not contacted.

## Supporting information

Supporting information.

Supporting information.

Supporting information.

Supporting information.

## Data Availability

Data used in this study is not publicly available. Data is available via reasonable request and study plan from the Institutional Review Board from the Pirkanmaa Hospital district.
