# Supplementary material for: Clinical outcomes after revision knee arthroplasty due to periprosthetic joint infection: A single‐centre study of 359 knees at a high‐volume centre with a minimum of one year follow‐up
Source: Knee Surg Sports Traumatol Arthrosc. 2025 Jul 7;33(11):3906–14. doi: 10.1002/ksa.12762 (PMC12582235; doi:10.1002/ksa.12762)
Supplement: Supplementary file 1 — Supporting information. [file KSA-33-3906-s003.docx]

**Supplementary file 1. Variables included in the global models before variable selection processes applied.**

1. Age
2. Sex
3. Diabetes Mellitus
4. ASA-score
5. C-reactive protein (serum)
6. Presence of fistula
7. Indication of the previous surgery (primary TKA / revision TKA)
8. Rheumatoid arthritis
9. Chronic kidney disease
10. Body mass index
11. Pathogen
12. Type of the revision (DAIR / one-stage revision / two-stage revision)
13. Type of the infection (early / acute hematogenous / chronic)
