# Supplementary material for: Clinical outcomes after revision knee arthroplasty due to periprosthetic joint infection: A single‐centre study of 359 knees at a high‐volume centre with a minimum of one year follow‐up
Source: Knee Surg Sports Traumatol Arthrosc. 2025 Jul 7;33(11):3906–14. doi: 10.1002/ksa.12762 (PMC12582235; doi:10.1002/ksa.12762)
Supplement: Supplementary file 2 — Supporting information. [file KSA-33-3906-s002.docx]

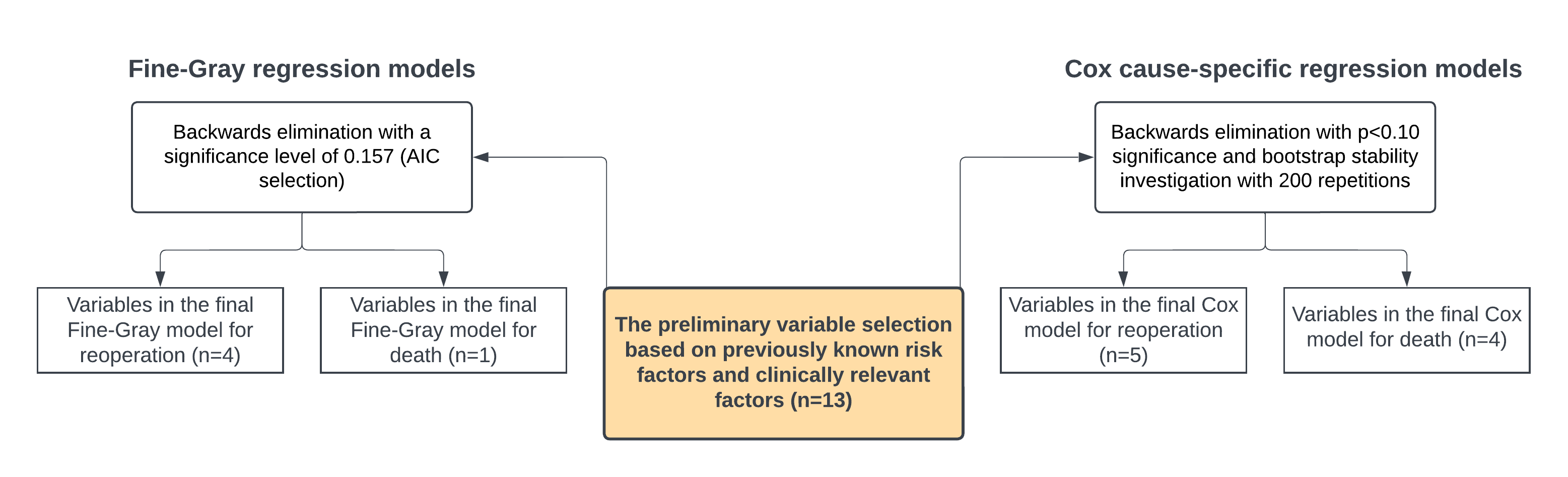


**Supplementary Figure 1.** Flow chart summarizing the predictor variable choices for Fine-Gray and Cox regression models.


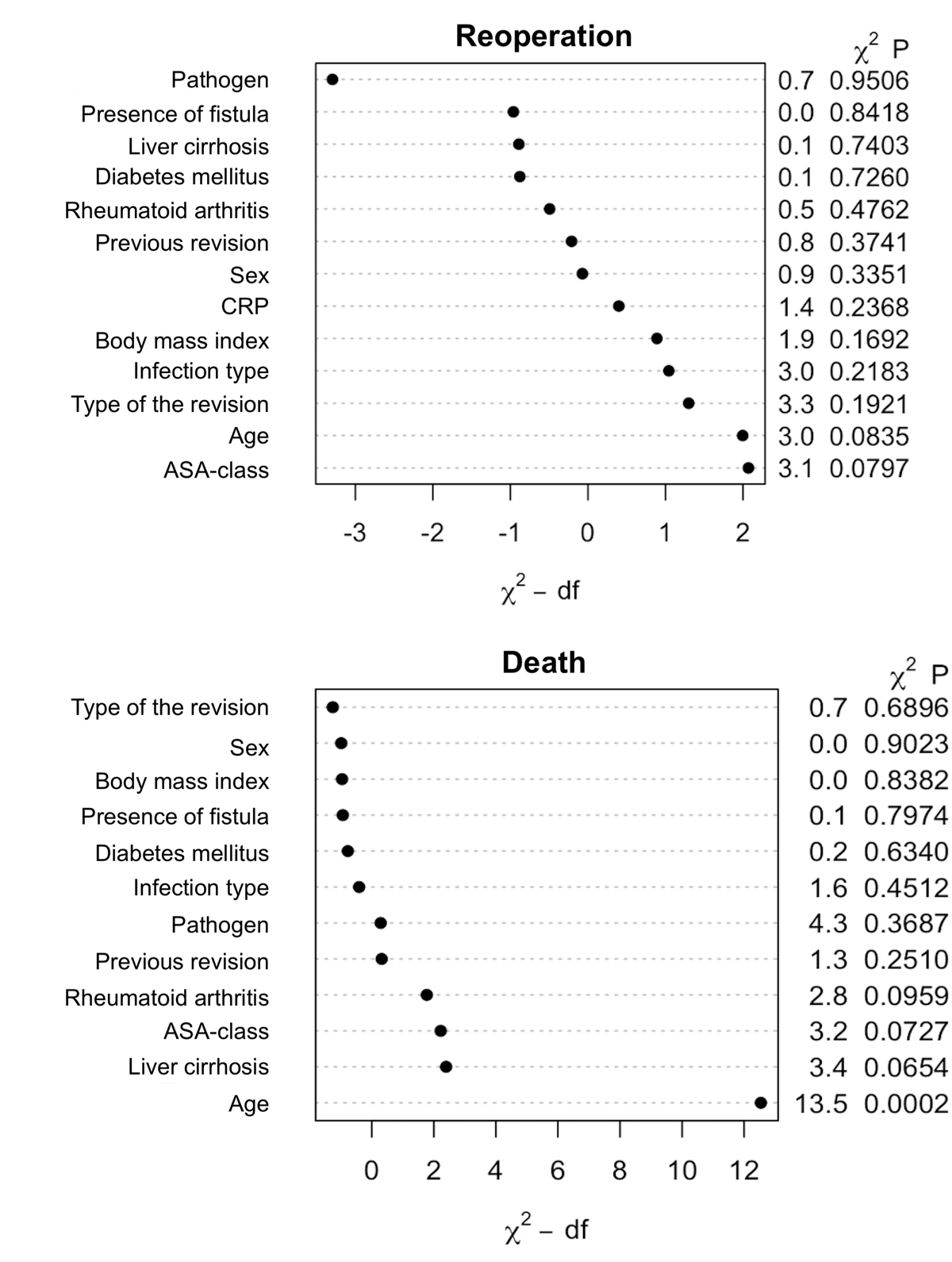


**Supplementary Figure 2.** The Chi-Squared regression coefficients for the predictors in the Cox regression models. The American Society of Anesthesiologists (ASA) class was the most important predictor of reoperation, and age was the most important predictor of death. ASA = American Society of Anesthesiology, DM = diabetes mellitus, BMI = body mass index, CCI = Charlson Comorbidity index.
