# Supplementary material for: Clinical outcomes after revision knee arthroplasty due to periprosthetic joint infection: A single‐centre study of 359 knees at a high‐volume centre with a minimum of one year follow‐up
Source: Knee Surg Sports Traumatol Arthrosc. 2025 Jul 7;33(11):3906–14. doi: 10.1002/ksa.12762 (PMC12582235; doi:10.1002/ksa.12762)
Supplement: Supplementary file 3 — Supporting information. [file KSA-33-3906-s004.docx]

| **Outcome** | **All revisions (n=359)** | | **DAIR  (n = 141)** | | **One-stage  (n = 98)** | | **Two-stage  (n = 120)** | |
| --- | --- | --- | --- | --- | --- | --- | --- | --- |
|  | N | *%* | N | *%* | N | *%* | N | *%* |
| **Tier 1: Infection control with no continued antibiotic therapy** | 164 | *45.7* | 69 | *48.9* | 55 | *56.1* | 40 | *33.3* |
| **Tier 2: Infection control with the patient on suppressive antibiotic therapy** | 3 | *0.8* | 1 | *0.7* | 2 | *2* | 0 | *0* |
| **Tier 3: Need for reoperation and/or revision and/or spacer retention** |  |  |  |  |  |  |  |  |
| 3A: Aseptic revision at >1 year from initiation of PJI treatment | 12 | *3.3* | 3 | *2.1* | 3 | *3.1* | 6 | *5* |
| 3B: Septic revision (including DAIR) at >1 year from initiation of PJI treatment | 18 *(17 )** | *5* | 6 *(33) ** | *4.3* | 4 *(0) ** | *4.1* | 8 *(13) ** | *6.7* |
| 3C: Aseptic revision at ≤1 year from initiation of PJI treatment | 3 | *0.8* | 0 | *0* | 1 | *1* | 2 | *1.7* |
| 3D: Septic revision (including DAIR) at ≤1 year from initiation of PJI treatment | 78 *(22) ** | *21.7* | 34 *(29) ** | *24.1* | 14 *(29) ** | *14.3* | 30 *(10) ** | *25* |
| 3E: Amputation, resection arthroplasty, or arthrodesis | 6 | *1.7* | 1 | *0.7* | 2 | *2* | 3 | *2.5* |
| 3F: Retained spacer | 3 | *0.8* | - | - | - | - | 3 | *2.5* |
| **Tier 4: Death** |  |  |  |  |  |  |  |  |
| 4A: ≤1 year from initiation of PJI treatment | 13 | *3.6* | 6 | *4.3* | 3 | *3.1* | 4 | *3.3* |
| 4B: >1 year from initiation of PJI treatment | 59 | *16.4* | 21 | *14.9* | 14 | *14.3* | 24 | *20* |

**Supplementary Table 1. Prosthetic Joint Infection Treatment Outcomes according to Musculoskeletal Infection Society Categorization scheme.** DAIR = debridement, antibiotics, and implant retention, *****=percentage of cases that are reinfected with the same initial organism.
