# Supplementary material for: Clinical outcomes after revision knee arthroplasty due to periprosthetic joint infection: A single‐centre study of 359 knees at a high‐volume centre with a minimum of one year follow‐up
Source: Knee Surg Sports Traumatol Arthrosc. 2025 Jul 7;33(11):3906–14. doi: 10.1002/ksa.12762 (PMC12582235; doi:10.1002/ksa.12762)
Supplement: Supplementary file 4 — Supporting information. [file KSA-33-3906-s001.docx]

| **Fine-Gray regression (n=274)** | | |
| --- | --- | --- |
|  | Adjusted sdHR (95% CI) | |
| **Subdistribution hazard ratios for reoperation^†^** | |  |
| CRP | 1.002 *(1.000–1.003)* | |
| Age | 0.98 *(0.96–1.00)* | |
| BMI | 1.04 *(1.00–1.07)* | |
| **Subdistribution hazard ratios for death** | |  |
| Age | 1.07 *(1.04–1.11)* | |

**Supplementary Table 2. Fine-Gray regression subdistribution hazard ratios for failure with 95% confidence intervals.** sdHR = subdistribution hazard ratio, CI = confidence interval, ASA = American Society of Anesthesiologists, BMI = body mass index, CRP = c-reactive protein. †=Type of the infection is adjusted for this model.
